# 1226. Two consecutive outbreaks of carbapenemase-producing enterobacteriaceae (CPE) in a pediatric hospital

**DOI:** 10.1093/ofid/ofac492.1058

**Published:** 2022-12-15

**Authors:** Meirav Mor, Sigalit Rozenfeld, Sarit Bitan, Haim Ben Zvi, Dalit Cohen, Itzhak Levy, Nurit Baruch, Efrat Bron-Harlev

**Affiliations:** Schneider Children's Medical Center of Israel; Tel Aviv University, Petach Tikva, Israel; Schneider Children's Medical Centerl in Israel, Petach Tikva, Tel Aviv, Israel; Schneider Children's Medical Centerl in Israel, Petach Tikva, Tel Aviv, Israel; Rabin Medical Center; Tel Aviv University, Petach Tikva, Tel Aviv, Israel; Schneider Children's Medical Center in Israel, Petach Tikva, Tel Aviv, Israel; Schneider Children's Medical Center in Israel; Tel Aviv University, Petach Tikva, Tel Aviv, Israel; Schneider Children's Medical Center in Israel, Petach Tikva, Tel Aviv, Israel; Schneider Children's Medical Center in Israel; Tel Aviv University, Petach Tikva, Tel Aviv, Israel

## Abstract

**Background:**

Carbapenemase-producing enterobacteriaceae (CPE) can cause hospital outbreaks with considerable health and economic implications. Intensive infection control measures are implemented to identify CPE carriers and contain outbreaks.

**Methods:**

We describe a CPE-NDM (New Delhi Metallo-beta-lactamase) hospital wide outbreak, in a tertiary care pediatric hospital, which evolved into a CPE-KPC (Klebsiella pneumoniae carbapenemase) outbreak before subsiding.

**Results:**

At 10/20/21 rectal screening for CPE was performed in patients in the NICU (neonatal intensive care unit) because of known CPE carriers hospitalized there. Multiple CPE carriers were discovered, and a hospital-wide outbreak of CPE-NDM was identified. All patients who were potential contacts of CPE carriers throughout the hospital were isolated and screened twice a week, as part of intensive infection control measures. Between 10/20/21 and 12/07/21 46 patients carrying CPE-NDM were discovered. Continued screening between 12/15/21 and 04/18/22 identified 81 additional patients carrying CPE, however the carbapenemases identified in 78 of them were KPC. No known CPE-KPC carrier was hospitalized at the beginning of the KPC outbreak. Surprisingly, in 6 of the patients carrying CPE-NDM, repeat screening done months later found the carriage shifted from CPE-NDM to CPE-KPC. The results were validated and analyzed at the National Infection Control Laboratory. No significant morbidity or mortality was observed due to CPE carriage.

CPE identification distribution by week and mechanism

**Figure:**
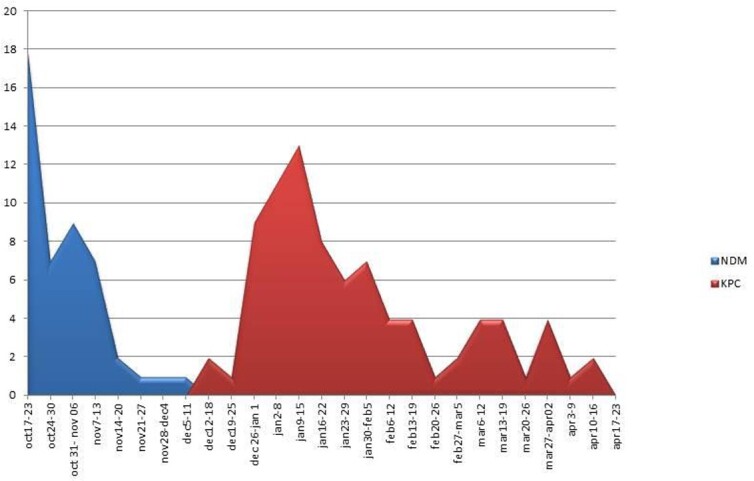

**Conclusion:**

We report an unusual sequence of two CPE outbreaks. We found no previous reports of consecutive outbreaks of CPE.

**Disclosures:**

**All Authors**: No reported disclosures.

